# In early rheumatoid arthritis, comorbidities do not explain the increased risk of failure to reach remission in patients with obesity

**DOI:** 10.1136/rmdopen-2025-005430

**Published:** 2025-04-15

**Authors:** Liselotte Tidblad, Anton Öberg Sysojev, Bénédicte Delcoigne, Lars Klareskog, Lars Alfredsson, Johan Askling, Helga Westerlind, Saedis Saevarsdottir

**Affiliations:** 1 Clinical Epidemiology Division, Department of Medicine Solna, Karolinska Institutet, Stockholm, Sweden; 2 Division of Rheumatology, Department of Medicine Solna, Karolinska Institutet, Stockholm, Sweden; 3 Institute of Environmental Medicine, Karolinska Institutet, Stockholm, Sweden; 4 Faculty of Medicine, University of Iceland, School of Health Sciences, Reykjavík, Iceland

**Keywords:** Arthritis, Rheumatoid, Epidemiology, Sweden, Methotrexate

## Abstract

**ABSTRACT:**

**Objective:**

To examine whether obesity and/or overweight are independently associated with an increased risk of remission failure in patients with early rheumatoid arthritis (RA), treated with methotrexate as first disease-modifying antirheumatic drug, or if the previously reported associations could be explained by underlying comorbidities and lifestyle factors.

**Methods:**

For patients included in the Epidemiological Investigation of Rheumatoid Arthritis (EIRA) study 2006–2018 initiating methotrexate monotherapy (n=1285), we captured data on body mass index, comorbidities and disease activity from EIRA and through linkage to nationwide Swedish clinical and quality registers. The primary outcome was failure to reach 28-joint Disease Activity Score (DAS28) remission at 3 and 6 months. Secondary outcomes included Boolean, Simplified Disease Activity Index and Clinical Disease Activity Index remission and their individual components. We estimated the relative risk (RR) of remission failure in patients with obesity and overweight compared with normal weight using modified Poisson regression, adjusting for potential confounders.

**Results:**

After 6 months, 64% (n=98/153) of patients with obesity, 52% (n=171/326) with overweight and 48% (n=210/433) with normal weight failed to reach DAS28 remission, with an RR of 1.33 (95% CI 1.14 to 1.55) for patients with obesity after adjustment for age and sex. The increased risk of remission failure in patients with obesity remained after further adjustment for seropositivity, educational level, smoking, alcohol use, physical activity, calendar period, glucocorticoid treatment and comorbidities (RR=1.27, 95% CI 1.08 to 1.50). No significant association was observed for patients with overweight. The results were similar for the secondary outcomes and after 3 months.

**Conclusion:**

Obesity is a risk factor for remission failure in early RA, independent of comorbid conditions.

**WHAT IS ALREADY KNOWN ON THIS TOPIC**

- Previous studies have shown that obesity is associated with remission failure in rheumatoid arthritis (RA). The reason for this remains unclear.
- To our knowledge, it has not been evaluated if the association could be explained by underlying comorbidities or lifestyle factors.

**WHAT THIS STUDY ADDS**

- In this study, patients with obesity and early RA were less likely to reach remission at 3 and 6 months after methotrexate start, compared with patients of normal weight.
- The associations remained after adjustments for sex, age, social and lifestyle factors as well as comorbid conditions.

**HOW THIS STUDY MIGHT AFFECT RESEARCH, PRACTICE OR POLICY**

- These results support that obesity is an independent risk factor for remission failure in early RA, irrespective of comorbid conditions.

## Introduction

 Obesity is linked to reduced quality of life, increased morbidity and a shortened life expectancy and represents an increasing challenge to global health.^1^,^4^ In rheumatoid arthritis (RA), previous studies have shown that patients with obesity have a higher RA disease activity and are less likely to reach remission compared with patients with normal weight.^5^,^10^ The reason for this remains unclear. Adipose tissue is known to have proinflammatory properties, and inflammatory parameters are elevated in patients with obesity, particularly women.^11^,^13^ Individuals with obesity also report higher levels of pain and a lower general assessment of their health.^14^,^16^

Several diseases are intimately linked with obesity, including type 2 diabetes, cardiovascular and respiratory diseases, certain malignancies and depression.^2 3 17^ Patients with established RA and concurrent comorbidities have been shown to have higher disease activity and be less responsive to treatment in several studies.^18^,^21^ In a recent study on remission in patients with early RA treated with methotrexate (MTX), we found that the overall comorbidity burden and certain comorbidity categories, including psychiatric, gastrointestinal, infectious and respiratory diseases, were associated with an increased risk of remission failure.^22^

Both obesity and comorbidities are thus important when studying RA remission, but to our knowledge, studies evaluating these factors in relation to each other are lacking. The aim of this present study was thus to investigate whether obesity and/or overweight are independently associated with an increased risk of remission failure in patients with early RA, treated with MTX in disease-modifying antirheumatic drug (DMARD) monotherapy, or if the previously reported associations could be explained by underlying comorbidities or lifestyle factors.

## Patients and methods

### Data sources

Our study population consisted of patients included in the Epidemiological Investigation of Rheumatoid Arthritis (EIRA) study, with data linked to nationwide Swedish registers (online supplemental table S1).^23^ EIRA is a population-based case–control study, covering the central and southern parts of Sweden, and includes patients ≥18 years old with newly diagnosed RA, fulfilling the 1987 American College of Rheumatology (ACR) and/or the 2010 European Alliance of Associations for Rheumatology (EULAR) criteria for RA (data from EIRA controls were not included in this study). Participants were included at RA diagnosis, at which the patients were asked to fill out a questionnaire covering questions on environmental exposures, socioeconomic and lifestyle factors as well as self-reported weight and height, which was the source for body mass index (BMI) data in the study. The participants were contacted via telephone if the questionnaires were incomplete. The questionnaire response rate was 93% for the cases.

Data from EIRA were linked to the Swedish Rheumatology Quality Register (SRQ), covering over 85% of all patients with RA in Sweden, in which information on disease activity and antirheumatic drugs is registered as part of the clinical routine at diagnosis and at follow-up visits.^24^ SRQ provided information on the start date for MTX (used as index date) and clinical information on disease activity (28 swollen joint count/tender joint count (SJC/TJC), erythrocyte sedimentation rate (ESR), C reactive protein (CRP), Patient Global Assessment (PGA) and pain on a visual analogue scale (VAS) of 0–100 mm).

The National Patient Register (NPR) contains International Classification of Diseases 10th Revision (ICD-10) diagnoses from hospital discharge and specialised outpatient care visits, and linkage to NPR was used to retrieve data on comorbidities. The Swedish Cancer Register was used for cancer diagnoses. Through linkage to the Prescribed Drug Register (PDR), information about prescribed and dispensed drugs was captured.

### Inclusion criteria

**Figure 1:**
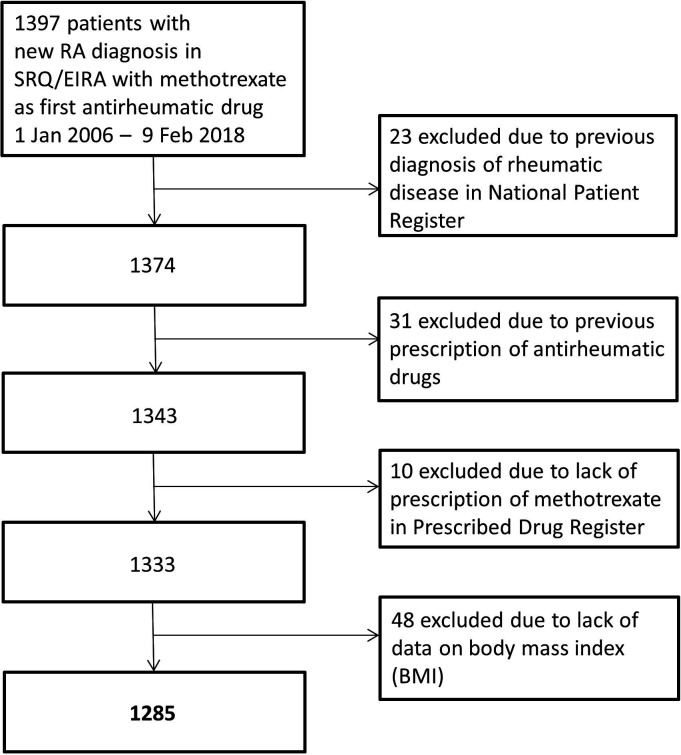
Flow chart showing selection of study population. EIRA, Epidemiological Investigation of Rheumatoid Arthritis; RA, rheumatoid arthritis; SRQ, Swedish Rheumatology Quality Register.

We included all patients from EIRA with registered initiation of a first ever MTX treatment between 1 January 2006 and 9 February 2018. Patients were excluded if diagnosis codes for a rheumatic disease had been registered in NPR more than 1 year before initiation of MTX or if there were registered dispensations of DMARDs according to the PDR within 18 months prior to start of MTX (figure 1 and online supplemental table S2). We further required that MTX (as registered in SRQ) also had been dispensed from a pharmacy, as well as available data on weight and height.

### Exposures and comorbidity data

Patients were divided into BMI categories: underweight: <18.5 kg/m^2^, normal weight: 18.5–24.9 kg/m^2^, overweight: 25–29.9 kg/m^2^ and obesity: ≥30 kg/m^2^. Data on educational level (≤9, 10–12, >12 years), physical activity during the previous year (active: moderate regular exercise or regular exercise/inactive: sedentary or moderate exercise),^25^ alcohol intake (≤13, 14–40, 41–88, ≥89 g/week) and smoking status (never/past/current) were also retrieved from EIRA. Information on serological status according to ICD codes, glucocorticoid treatment at MTX initiation (yes/no) and calendar period of MTX start (2006–2014, 2015–2018) was captured from SRQ.

We captured 31 different comorbid conditions, grouped into 10 comorbidity categories (cardiovascular, non-cardiac vascular, malignant, endocrine, gastrointestinal, infectious diseases, chronic kidney disease, neurological, psychiatric and respiratory; see table 1 and online supplemental table S3), as previously described.^26 27^ For infectious diseases, only ICD-10 codes that were registered in conjunction with hospitalisations with an infectious disease as the main diagnosis were used. Information about comorbidities was captured from the date of MTX initiation and 5 years prior. For some comorbid conditions, for which patients are mainly managed in primary care and for which there are specific treatments (eg, antidepressants, oral antidiabetics, insulin, lipid-lowering drugs, thyroid and antidementia drugs), we used drug dispensations from PDR during 18 months up until the MTX initiation as an extended assessment of comorbidities. The Anatomical Therapeutic Chemical classification and ICD-10 codes that were used are shown in online supplemental table S3.

**Table 1:** Comorbidity categories with the corresponding diagnoses

Cardiovascular	Ischaemic heart disease
	Acute coronary syndrome
	Heart failure
	Atrial fibrillation
Non-cardiac vascular	Peripheral vascular diseases
	Lipid-lowering drugs
	Stroke
	Transient ischaemic attack
	Thromboembolic venous disease
	Hypertension
Respiratory	Chronic obstructive pulmonary disease
	Chronic interstitial pulmonary disease
	Asthma
Gastrointestinal	Inflammatory bowel disease
	Oesophageal, gastric and duodenal diseases
	Biliary disease
	Chronic liver disease
Psychiatric	Depression
	Antidepressant drugs
	Anxiety disorder
	Psychosis
	Dementia (antidementia drugs)
Chronic kidney	Chronic kidney disease
Infectious	Hospitalisation due to infectious diseases
Endocrine	Type 1 diabetes (insulin)*
	Type 2 diabetes (glucose-lowering drugs)†
	Thyroid disease (thyroid and antithyroid preparations)
Malignant	Cancer
Neurological	Parkinson’s disease
	Multiple sclerosis
	Epilepsy
	Polyneuropathies and nerve, nerve root and plexus disorders

* To be considered having type 1 diabetes, participants had to have an E10 diagnosis and previous prescription of insulin, without E11 diagnosis and any previous prescription of glucose-lowering drugs.

† To be considered having type 2 diabetes, participants had to have either an E11 diagnosis or a prescription of glucose-lowering drugs without a previous E10 diagnosis. For details on International Classification of Diseases 10th rRevision (ICD-10) codes and Anatomical Therapeutic Chemical (ATC) classification codes, see supplemental table S3online supplemental table S3.

The Rheumatic Disease Comorbidity Index (RDCI) was used as a measure of overall comorbidity burden. RDCI is a weighted index that covers lung disease, cardiovascular disease, hypertension, fractures, depression, diabetes, cancer and ulcer/stomach problems (online supplemental table S4).^28^,^30^ We used the same ICD codes as for the comorbidity categories along with ICD codes for fractures (online supplemental table S3) to calculate the RDCI.

### Outcomes and follow-up visits

The primary outcome was 28-joint Disease Activity Score (DAS28) remission, defined as DAS28 <2.6 or DAS28-CRP <2.4. Clinical Disease Activity Index (CDAI) remission, Simplified Disease Activity Index (SDAI) remission, ACR/EULAR Boolean remission, EULAR good response (compared with moderate/no response) and no swollen joints (using 28 joints) were analysed as secondary outcomes. Definitions of the remission outcomes are summarised in online supplemental table S5.^31^,^35^ Additional secondary outcome measures were the components of DAS28/DAS28-CRP (cut-offs in parentheses: PGA (VAS>20 mm), TJC (>1), SJC (>1), CRP (>10 mg/L), ESR (>20 mm/hour for women/>15 mm/hour for men), as well as pain (VAS>20 mm)).

We assessed the outcomes at 3 and 6 months after MTX initiation with data collected as previously described.^36^ In brief, visits registered in SRQ 31–149 days after the index date were used to identify the 3-month visit, and visits in SRQ 150–269 days after the index date for the 6-month visit. If there were several visits registered within the same time period, we used the visit that (1) had the highest completeness of the clinical data and (2) was closest in time to the target date (ie, 90 and 180 days, respectively), and (3) in case MTX was discontinued, we used the last visit that occurred during treatment with MTX monotherapy.

### Statistical analysis

Modified Poisson regression was used to evaluate the association between the exposure of the three different BMI categories and the primary outcome (failure to reach DAS28 remission) as well as multiple secondary outcomes (failure to reach Boolean, CDAI, SDAI remission, EULAR response, no swollen joints). We used three different models: (1) crude: adjusted for sex and age, (2) additionally adjusted for serological status, glucocorticoid treatment at MTX initiation, educational level, smoking, alcohol use, physical activity and calendar period of MTX start, and (3) fully adjusted: as in (2) and additionally adjusted for the 10 individual comorbidity categories. We used the same three models for analysing the association of BMI categories and DAS28 remission failure with stratification according to sex, serological status and smoking. We also performed an analysis comparing patients with obesity versus non-obesity.

Modified Poisson regression was also used to evaluate the association between the 10 individual comorbidity categories (cardiovascular, non-cardiac vascular, malignant, endocrine, gastrointestinal, infectious, chronic kidney disease, neurological, psychiatric and respiratory) and DAS28 remission failure, using the same three models, separately within each of the BMI categories (normal weight, overweight, obese) in order to evaluate whether the effect of comorbidities was only observed in certain BMI categories. Furthermore, modified Poisson regression was used to evaluate the effect of BMI categories on the individual remission measure components (PGA, TJC, SJC, CRP, ESR, as well as VAS pain) using the same three models. All assessments were made at 3 and 6 months after MTX initiation.

To avoid issues relating to exposure categories with very few observations, we chose not to perform the regression analysis whenever any exposure category contained five or fewer individuals.

## Results

### Patient characteristics

The study group consisted of 1285 patients with early RA who received MTX as the first DMARD in monotherapy (figure 1). Of these, 70% were female and 66% seropositive. The median age for all patients was 58 (IQR 47–66) years. Nearly half of the patients (n=598, 47%) were classified as having normal weight, 35% (n=453) overweight and 17% (n=217) obesity. Half of the patients (n=633, 49%) had one or more of the reported comorbid conditions, ranging from 40% among patients with normal weight, 54% among patients with overweight to 65% among patients with obesity (table 2). Due to a low proportion of patients with underweight (1%) in our cohort, this subgroup was not included in any further analysis. Further details on the baseline characteristics of patients across BMI categories are provided in table 2.

**Table 2:** Baseline characteristics of the 1285 Swedish patients with early RA receiving methotrexate monotherapy during 2006–2018, stratified into BMI categories

	Overalln=1285	Normal weightn=598	Overweightn=453	Obesityn=217
Women, n (%)	894 (70)	448 (75)	273 (60)	156 (72)
Seropositive, n (%)	842 (66)	381 (64)	301 (66)	148 (68)
Age: 18–49 years	381 (30)	208 (35)	108 (24)	58 (27)
Age: 50–74 years	839 (65)	363 (61)	317 (70)	150 (69)
Age: ≥75 years	65 (5)	27 (5)	28 (6)	9 (4)
RDCI: 0, n (%)	980 (76)	501 (84)	326 (72)	137 (63)
RDCI: 1, n (%)	164 (13)	58 (10)	62 (14)	44 (20)
RDCI: 2, n (%)	73 (6)	20 (3)	37 (8)	15 (7)
RDCI: ≥3, n (%)	68 (5)	19 (3)	28 (6)	21 (10)
Cardiovascular, n (%)	72 (6)	23 (4)	29 (6)	20 (9)
Non-cardiac vascular, n (%)	270 (21)	83 (14)	116 (26)	69 (32)
Malignant, n (%)	47 (4)	23 (4)	12 (3)	12 (6)
Endocrine, n (%)	201 (16)	64 (11)	81 (18)	53 (24)
Gastrointestinal, n (%)	50 (4)	15 (3)	22 (5)	13 (6)
Infectious, n (%)	63 (5)	27 (5)	22 (5)	14 (6)
Chronic kidney disease, n (%)	7 (1)	<5	<5	<5
Neurological, n (%)	78 (6)	36 (6)	26 (6)	14 (6)
Psychiatric, n (%)	172 (13)	69 (12)	60 (13)	40 (18)
Respiratory, n (%)	49 (4)	9 (2)	28 (6)	11 (5)
Any comorbidity, n (%)	633 (49)	238 (40)	246 (54)	140 (65)

Normal weight: BMI 18.5–24.9 kg/m2, overweight: BMI 25–29.9 kg/m2, obesity: BMI≥30 kg/m2. Underweight (BMI<18.5 kg/m2) only consisted of 17 patients (1%) and werewas omitted from the table. Missing data on serological status: 18 (1%).

BMI, body mass index; RA, rheumatoid arthritis; RDCI, Rheumatic Disease Comorbidity Index.

### Proportions failing to reach DAS28 remission

Data on DAS28 remission were available for 1088 patients (85%) at 3 months and 928 patients (72%) at 6 months after MTX initiation (the proportion of missing data was similar across BMI categories, data not shown). Among these, 54% (588/1088) failed to reach DAS28 remission at 3 months and 53% (488/928) at 6 months. By 3 months, 62% (114/185) of patients with obesity and 53% (205/384) of patients with overweight did not reach DAS28 remission, compared with 52% (261/505) of those with normal weight. Similarly, by 6 months, 64% (98/153) of patients with obesity, 52% (171/326) with overweight and 48% (210/433) with normal weight failed to reach DAS28 remission (online supplemental table S6).

### Impact of obesity and comorbidities on risk of failure to reach DAS28 remission

**Figure 2:**
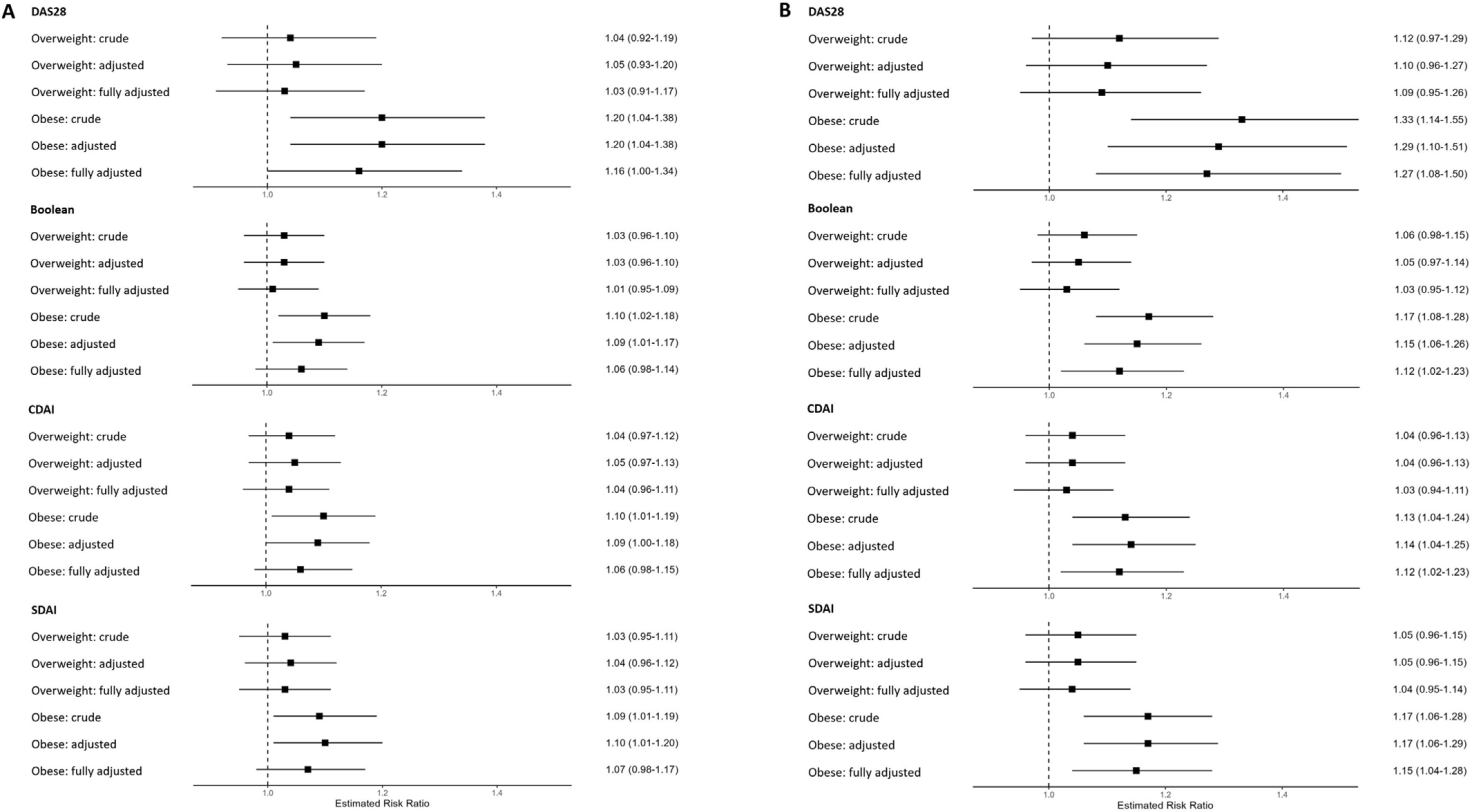
Relative risk of patients with overweight and obesity and early rheumatoid arthritis (RA) of not reaching remission compared with patients with normal weight, estimated by modified Poisson regression. (**A**) At 3 months after RA diagnosis. (**B**) At 6 months after RA diagnosis. Normal weight: BMI 18.5–24.9 kg/m^2^ (reference), overweight: BMI 25–29.9 kg/m^2^, obese: BMI≥30 kg/m^2^. Crude: adjusted for sex and age. Adjusted: adjusted for serological status, glucocorticoid treatment at methotrexate initiation (yes/no), educational level, smoking, alcohol use, physical activity and calendar period of methotrexate start. Fully adjusted: adjusted+the individual comorbidity categories. Numbers are shown in online supplemental tables S7 and S10. BMI, body mass index; CDAI, Clinical Disease Activity Index; DAS28, 28-joint Disease Activity Score; SDAI, Simplified Disease Activity Index.

Patients with obesity had an increased risk of DAS28 remission failure at 3 months compared with patients with normal weight after adjustment for age and sex (relative risk (RR)=1.20, 95% CI 1.04 to 1.38) (crude model). After further adjustments for seropositivity, educational level, smoking, alcohol use, physical activity, calendar period and glucocorticoid treatment (adjusted model), the RR remained at 1.20 (95% CI 1.04 to 1.38), and when adjustments for comorbidities were added (full model), the RR was 1.16 (95% CI 1.00 to 1.34). After 6 months, the RR of remission failure for the patients with obesity was 1.33 (95% CI 1.14 to 1.55) in the crude model and 1.29 (95% CI 1.10 to 1.51) in the adjusted model. The increased risk of remission failure in patients with obesity remained in the fully adjusted model (RR=1.27, 95% CI 1.08 to 1.50). No statistically significant associations were observed for the patients with overweight, although the RR of remission failure was in the same direction as for patients with obesity in all three models (figure 2, online supplemental table S7).

### Analyses on failure to reach DAS28 remission in subgroups

Analyses stratified by sex demonstrated that the increased risk of remission failure among patients with obesity was only seen among women at both 3 months (RR=1.26, 95% CI 1.08 to 1.49) and 6 months (RR=1.36, 95% CI 1.13 to 1.62, all results from the fully adjusted model), while obesity had no effect on the risk of remission failure among men (3 months: RR=0.85, 95% CI 0.61 to 1.20, 6 months: RR=1.08, 95% CI 0.75 to 1.55). In the seropositive RA subgroup, patients with obesity had a higher risk of DAS28 remission failure compared with patients with normal weight at 6 months (RR=1.25, 95% CI 1.02 to 1.53), and we noted a similar pattern in seronegative patients (RR=1.23, 95% CI 0.93 to 1.64). Among smokers, both patients with overweight (RR=1.24, 95% CI 1.03 to 1.49) and obesity (RR=1.36, 95% CI 1.11 to 1.67) were at higher risk of not reaching DAS28 remission at 6 months compared with patients of normal weight (online supplemental table S8). We found no interaction between BMI and sex, serological status or smoking (data not shown).

We also evaluated whether the risk of DAS28 remission failure associated with certain comorbidity categories was only observed within some of the BMI categories. Several comorbidity categories affected the risk of remission failure, especially among the patients with overweight at 3 months (malignant, neurological and psychiatric diseases), whereas there were fewer findings among patients with obesity. The precision in these subgroup analyses was, however, limited due to the smaller sample size (online supplemental table S9).

### Secondary remission measures

The higher risk of failure to reach remission among patients with obesity compared with patients with normal weight was also observed for several of the secondary remission measures, including Boolean, CDAI and SDAI remission, with somewhat stronger associations at 6 months than 3 months of follow-up. These associations remained after adjustment for comorbidities and other potential confounders (figure 2 and online supplemental table S10). There was an increased risk of not reaching EULAR response at 3 months, but not for no swollen joints at 3 or 6 months (online supplemental table S10). In line with the analyses of the primary outcome, no significant results were seen for patients with overweight compared with normal weight.

### Remission measure components

**Figure 3:**
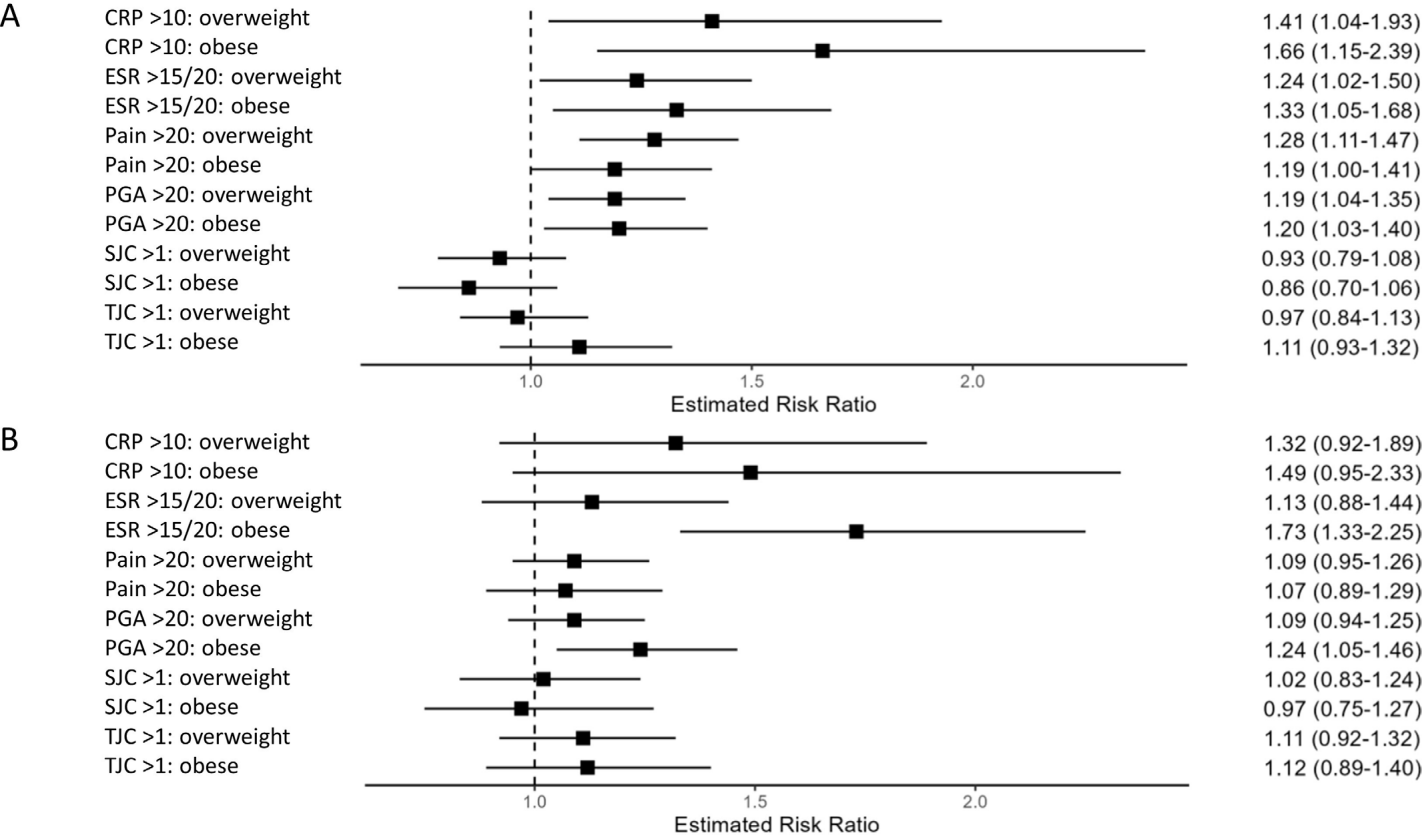
Relative risk of patients with overweight and obesity and early rheumatoid arthritis (RA) of scoring over the cut-off of the 28-joint Disease Activity Score (DAS28) components and visual analogue scale (VAS) pain compared with patients with normal weight, estimated by modified Poisson regression. (**A**) At 3 months after RA diagnosis. (**B**) At 6 months after RA diagnosis. Normal weight: BMI 18.5–24.9 kg/m^2^ (reference), overweight: BMI 25–29.9 kg/m^2^, obese: BMI≥30 kg/m^2^. Results from the fully adjusted model are shown in the figure: adjusted for serological status, glucocorticoid treatment at methotrexate initiation, educational level, smoking, alcohol use, physical activity, calendar period of methotrexate start and the individual comorbidity categories. All models and numbers are shown in online supplemental table S11. BMI, body mass index; CRP, C reactive protein (mg/L); ESR, erythrocyte sedimentation rate (mm) (ESR>20 mm/hour for women/>15 mm/hour for men); PGA, Patient Global Assessment in VAS; SJC, swollen joint count (28 joints); TJC, tender joint count (28 joints).

Among patients with overweight and obesity, there was an increased risk of having a systemic inflammation at follow-up, as assessed by CRP and ESR, which was significant at 3 months of follow-up for CRP elevation (>10 mg/L) and ESR (>20 mm/hour in women and >15 mm/hour in men) compared with patients with normal weight. At 6 months, there was an increased risk of ESR elevation among patients with obesity when compared with normal weight. The increased risk of not reaching disease control among patients with overweight and obesity was also seen for subjective measures at 3 months, that is, both pain (VAS>20 mm) and PGA (VAS>20 mm), but at 6 months only for PGA among the patients with obesity. No statistically significant associations between obesity or overweight and persistent joint inflammation (SJC>1) or tenderness (TJC>1) at follow-up were observed (figure 3, online supplemental table S11).

## Discussion

To our knowledge, this is the first study evaluating whether the increased risk of remission failure among patients with obesity and early RA can be explained by underlying comorbidities or lifestyle habits. Our findings support that obesity is an independent risk factor for remission failure, with consistent results across several remission measures and for most objective and subjective components of the composite disease activity measures. However, the association between obesity and remission failure did not extend to overweight.

Several studies have shown that obesity is associated with remission failure in RA.^5^,^1037^ As obesity is also linked to a number of comorbidities^2 3^ and lifestyle factors, one hypothesis would be that the increased risk of remission failure could be explained by these underlying factors. We adjusted for several common comorbidities and possible confounders, but the association to obesity still remained. The elevated risk of remission failure among patients with obesity, in particular women and smokers, is in line with several previous studies.^5^,^938 39^

Composite measures of disease activity (eg, DAS28, CDAI and SDAI) rely on patient-reported outcome measures and might also be affected by other factors that are not representing ‘true’ RA disease activity as much as characteristics of the individual. Increased occurrence of pain conditions and worse perceived health have also been observed in individuals with obesity in the general population.^14^,^16^ It is possible that subjective parameters of the remission measures could represent factors unrelated to the RA disease activity. Otherwise healthy people with high BMI have also been shown to have increased levels of inflammatory parameters.^12 13^ An association between high BMI and elevated CRP and/or ESR has also been found in patients with established RA, especially women.^13 19 40^Adipocytes produce adipokines and proinflammatory cytokines and can thereby lead to a chronic low-grade inflammation, not confined only to the adipose tissue.^41^ Adipokines are also thought to be modulators of different immune cells as well as local cells in synovial tissue, cartilage and bone.^11^ Levels of adipokines have been shown to be elevated in both plasma and synovial fluid in patients with RA.^42 43^ There are thus many potential mechanisms, on different levels, whereby obesity could affect RA disease activity. In this study, patients with overweight and obesity were not at increased risk of having SJC/TJC>1 compared with patients with normal weight, supporting that the remission failure might not be due to actual arthritic activity.

The study has some limitations, including that weight and height were self-reported. We lacked detailed information on dose adjustments of glucocorticoid use at 3 and 6 months and therefore only adjusted for glucocorticoid use at MTX initiation (in the adjusted and fully adjusted models). Lower use or shorter treatment period with glucocorticoids in patients with some comorbidities, including concurrent type 2 diabetes, might affect the likelihood of remission in our study. In subanalyses of comorbidity categories stratified by BMI categories, certain strata contained very few observations which limited the possibility for analysis, and in other instances led to some imprecision around the point estimates. Furthermore, the comorbidity data were mainly collected from specialised care, which could have excluded milder comorbid conditions that were followed in primary care. In the current study, we attempted to compensate for this by adding prescribed drugs, as an additional way of finding comorbidities with a specific treatment, for example, antidiabetic drugs and antidepressants.

Strengths of our study include the population-based design and the included adjustment for multiple potential confounders as well as comorbidities. Comorbidity data were collected through registers, which reduces the risk of recall bias. Furthermore, since we only included MTX-treated patients with early RA, we could compare remission rates without possible confounding due to differences in antirheumatic treatments and disease durations.

Taken together, our study confirms the previously described observation of an increased risk of remission failure among patients with obesity and early RA and extends this observation by demonstrating that this association could not be explained by the assessed comorbid conditions or lifestyle habits among patients with obesity. The association could neither be explained by specific subjective (TJC, PGA, VAS pain) nor by objective (SJC, CRP, ESR) components of the remission metrics. Whether the driving factor is an increased disease activity due to the RA disease itself, a worse perceived health and higher inflammation among patients with obesity, or both, remains to be determined. Nevertheless, our findings support the notion that rheumatologists have to be careful when assessing disease activity in this patient group. Whether weight loss can increase the likelihood of ‘true’ RA-specific remission and improve outcome and general health among patients with obesity and RA was outside the scope of this study, but remains an attractive hypothesis.

## Supplementary material

10.1136/rmdopen-2025-005430 online supplemental file 1

## Data Availability

Data are available upon reasonable request.
