# Supplementary material for: In early rheumatoid arthritis, comorbidities do not explain the increased risk of failure to reach remission in patients with obesity
Source: RMD Open. 2025 Apr 15;11(2):e005430. doi: 10.1136/rmdopen-2025-005430 (PMC12001363; doi:10.1136/rmdopen-2025-005430)
Supplement: online supplemental file 1 [file rmdopen-11-2-s001.docx]

Supplementary material

**Supplemental table S1.** List of registers used

| **Register** | **Description** |  |
| --- | --- | --- |
| **Epidemiological Investigation of Rheumatoid Arthritis (EIRA)** | Founded in 1996 at the Karolinska Institute. Collaborative  population-based case-control study of newly diagnosed cases with RA,  with main focus on risk factors for RA development. | |
| **National Cancer Register** | Founded in 1958. Covers the whole population. Newly | |
|  | detected cancer (diagnosed at clinical, morphological or | |
|  | other laboratory examinations or autopsies) are | |
|  | compulsory to report. | |
| **National Patient Register (NPR)** | Started in the 1960s. Mandatory participation from all | |
|  | counties since 1984. Covers all in-patient care in Sweden  since 1987. Since 2001 it also covers out-patient visits | |
|  | from specialized care from both private and public | |
|  | care-givers. Primary health care is not included. | |
| **National Prescribed Drug Register (PDR)** | Started in July 2005. Contains all prescribed drugs | |
|  | dispensed at pharmacies in Sweden. | |
| **Swedish Rheumatology Quality Register (SRQ)** | Founded in 1995. Covers information on diagnosis/ | |
|  | inclusion date, symptom duration, disease activity and | |
|  | treatment. | |
| **Total Population Register (TPR)** | Started in 1968 by Statistics Sweden (SCB). Covers | |
|  | information on the whole population concerning birth,  death, area of residence, immigration, emigration etc. |  |

**Supplemental table S2.** ICD-10 and ATC codes used for exclusion of patients.*

| \| **Diagnoses** \| **ICD-10 codes** \| \| --- \| --- \| \| Rheumatoid arthritis \| M05, M06.0, M06.2-M06.3, M06.8-M06.9, M12.3 \| \| Other arthritis \| M13 \| \| Psoriatic arthritis \| L40.5, M07.0, M07.1, M07.3 \| \| Ankylosing spondylitis \| M45 \| \| Juvenile arthritis \| M08-M09 \| \| Inflammatory spondylopathies \| M46.0-M46.1, M46.8-M46.9 \| \| SLE \| M32.0-M32.1, M32.8-M32.9 \| \| PMR \| M35.3 \| | | |  |
| --- | --- | --- | --- | --- | --- | --- | --- | --- | --- | --- | --- | --- | --- | --- | --- | --- | --- | --- | --- | --- | --- |
| Drugs | **ATC codes** |  |  |
| Abatacept | L04AA24 |  |  |
| Adalimumab | L04AB04 |  |  |
| Anakinra | L04AC03 |  |  |
| Apremilast | L04AA32 |  |  |
| Baricitinib | L04AA37 |  |  |
| Belimumab | L04AA26 |  |  |
| Canakinumab  Certolizumab pegol | L04AC08  L04AB05 |  |  |
| Etanercept | L04AB01 |  |  |
| Golimumab | L04AB06 |  |  |
| Chloroquine | P01BA01 |  |  |
| Leflunomide | L04AA13 |  |  |
| Methotrexate | L04AX03 |  |  |
| [Sodium aurothiomalate](https://www.whocc.no/atc_ddd_index/?code=M01CB01) | M01CB01 |  |  |
| Hydroxychloroquine | P01BA02 |  |  |
| Auranofin | M01CB03 |  |  |
| Secukinumab | L04AC10 |  |  |
| Sulfasalazine | A07EC01 |  |  |
| Tocilizumab | L04AC07 |  |  |
| Tofacitinib | L04AA29 |  |  |
| Ustekinumab | L04AC05 |  |  |

*Patients were excluded if they had any of these diagnoses registered >12 months before the index date/methotrexate start or any of these drugs prescribed >1 month before the index date/methotrexate start in SRQ (Swedish Rheumatology Quality register). ICD-10, International Classification of Diseases, 10^th^ revision;

ATC, Anatomical Therapeutic Chemical Classification; SLE, systemic lupus erythematosus; PMR, polymyalgia rheumatica.

**Supplemental table S3.** Comorbidity categories and diagnoses with corresponding ICD-10

and/or ATC codes used to capture each comorbidity.

| **Comorbidity categories** | **Diagnoses** | **ICD-10 codes** | **ATC codes** |
| --- | --- | --- | --- |
| **Cardiovascular** | Ischaemic heart disease | I20-I25 |  |
|  | Acute coronary syndrome | I200, 121-122 |  |
|  | Heart failure | I50 |  |
|  | Atrial fibrillation | I48 |  |
| **Non-cardiac vascular** | Peripheral vascular diseases | I65-I66, I70-I72, I739, I74 |  |
|  | Lipid lowering drugs |  | C10 |
|  | Stroke | I60-I64 |  |
|  | Transient ischaemic attack | G45 |  |
|  | Thromboembolic venous disease | I26, I80-I82 |  |
|  | Hypertension | I10-I15 |  |
| **Respiratory** | Chronic obstructive pulmonary disease | J43-J44 |  |
|  | Chronic interstitial pulmonary disease | J84 |  |
|  | Asthma | J45 |  |
| **Gastrointestinal** | Inflammatory bowel disease | K50-K51 |  |
|  | Esophageal, gastric and duodenal disease | K20-K23, K25-K27, K29 |  |
|  | Biliary disease | K80-K81 |  |
|  | Chronic liver disease | K70-K76 |  |
| **Psychiatric** | Depression | F32-F33 |  |
|  | Antidepressant drugs |  | N06A |
|  | Anxiety disorder | F40-F41, F43 |  |
|  | Psychosis | F20-F29 |  |
|  | Dementia (antidementia drugs) | F00-F03, G30-G31 | N06D |
| **Chronic kidney** | Chronic kidney disease | I12-I13, N03-N05, N07, |  |
|  |  | N18, Q61 |  |
| **Infectious** | Hospitalization due to infectious diseases | A00-B99, G00-G02, G042 |  |
|  |  | G05-G07, H66-H67, J00-J22, J32, J340, J36, J838,  J390-J391, J85-J86, L00-L08, M00-M01, M462-M465, M86, N10, N300, N390 |  |
| **Endocrine** | Type1 diabetes (insulin)* | E10 | A10A |
|  | Type 2 diabetes (glucose-lowering drugs)Ɨ | E11 | A10B |
|  | Thyroid disease (thyroid and antithyroid preparations) | E038-E039, E050-E053, E055-E059 | H03A, H03B |
| **Malignant** | Cancer | C00-C97, D00-D09 |  |
| **Neurological** | Parkinson's disease | G20 |  |
|  | Multiple sclerosis | G35 |  |
|  | Epilepsy | G40-G41 |  |
|  | Polyneuropathies and nerve, nerve root | G50-G59, G60-G65 |  |
|  | and plexus disorders |  |  |
| **Fractures**ǂ | Fractures of hip/spine/leg | M485, M489, M800A,  M800E-F, M800J-K, M843E-G, S120-S127, S220-S221, S32, T021, T08, S72, S821-S824, S827 |  |

Comorbidities were captured 5 years before the initiation of methotrexate. *For considered as having type 1 diabetes you had to have the E10 diagnosis, no E11 diagnosis, prescription of insulin and no prescription of glucose-lowering drugs. ƗFor considered as having type 2 diabetes you had to have the E11 diagnosis or prescription of glucose-lowering drugs and no E10 diagnosis. ǂInformation on fractures was only used for the Rheumatic Disease Comorbidity Index. ICD-10, International Classification of Diseases 10^th^ revision; ATC, Anatomical Therapeutic Chemical Classification.

**Supplemental table S4.** Rheumatic Disease Comorbidity Index.

| **Comorbidity groups** | Lung disease  Heart Attack  Other Cardiovascular disease  Stroke  Hypertension  Diabetes  Fracture (of hip/spine/leg)  Depression  Cancer  Gastrointestinal ulcer  Other stomach problems |
| --- | --- |
| **Formula** | 2 x lung disease + [2 x (heart attack, other cardiovascular disease, or stroke), or 1 x hypertension] + fracture + depression + diabetes + cancer + (ulcer or stomach problem) |

Table from: Aslam F, Khan NA. Tools for the Assessment of Comorbidity Burden in Rheumatoid Arthritis. *Frontiers in Medicine* 2018;5(39), adapted by data from: England BR, Sayles H, Mikuls TR, Johnson DS, Michaud K. Validation of the rheumatic disease comorbidity index. *Arthritis Care Res (Hoboken)* (2015) 67(6):865-72.

**Supplemental table S5.** Definitions of outcome measures

| \| Outcome \| Definition \| \| --- \| --- \| \|  \| **Remission** \| \| DAS28-remission \| DAS28 <2.6 or DAS28-CRP <2.4 \| \| ACR/EULAR Boolean remission \| TJC28 ≤1, SJC28≤1, PGA (0-10) ≤1 and CRP ≤1 \| \| SDAI remission \| SDAI ≤3.3 \| \| CDAI remission \| CDAI ≤2.8 \| \| No swollen joints \| SJC28 = 0 \| \|  \| **Response** \| \| EULAR response \| Either fulfilling a. or b. \| \|  \| a. DAS28_0 - DAS28_x ≥1.2 and DAS28_x ≤3.2 \| \|  \| b. DAS28-CRP_0 - DAS28-CRP_x ≥1.2 and DAS28_x ≤3.2 \| \|  \| where _0 denotes the start of treatment and _x denotes the month for evaluation \| |
| --- | --- | --- | --- | --- | --- | --- | --- | --- | --- | --- | --- | --- | --- | --- | --- | --- | --- | --- | --- | --- | --- | --- | --- | --- |

Table adapted from Westerlind et al., Remission, response, retention and persistence to treatment with disease-modifying agents in patients with rheumatoid arthritis: a study of harmonised Swedish, Danish and Norwegian cohorts. RMD Open. 2023 Sep;9(3):e003027, supplemental table S2.

DAS28, disease activity score (28 joints); CRP, C-reactive protein (mg/dL); SDAI, simplified disease activity index; CDAI, clinical disease activity index; ACR/EULAR, American College of Rheumatology/European Alliance of Associations for Rheumatology; TJC, tender joint count (28 joints); SJC, swollen joint count (28 joints); PGA, patient global assessment.

**Supplemental table S6.** Proportions not reaching DAS28-remission at 3 and 6 months after initiating methotrexate monotherapy in early RA

|  | Not reaching DAS28-remission at 3 months/n with data on remission | Not reaching DAS28-remission at 6 months/n with data on remission |
| --- | --- | --- |
| OVERALL, n (%) | 588/1088 (54) | 488/928 (53) |
| Normal weight, n (%) | 261/505 (52) | 210/433 (48) |
| Overweight, n (%) | 205/384 (53) | 171/326 (52) |
| Obese, n (%) | 114/185 (62) | 98/153 (64) |
| RDCI: 0, n (%) | 431/828(52) | 378/712 (53) |
| RDCI: 1, n (%) | 83/142 (58) | 62/117 (53) |
| RDCI: 2, n (%) | 34/60 (57) | 18/50 (36) |
| RDCI: ≥3, n (%) | 40/58 (69) | 30/49 (61) |
| Cardiovascular, n (%) | 39/62 (63) | 26/48 (54) |
| Non-cardiac vascular, n (%) | 135/234 (58) | 95/182 (52) |
| Malignant, n (%) | 19/41 (46) | 15/31 (48) |
| Endocrine, n (%) | 94/170 (55) | 85/148 (57) |
| Gastrointestinal, n (%) | 28/45 (62) | 18/38 (47) |
| Infectious, n (%) | 32/52 (62) | 17/45 (38) |
| Chronic kidney disease, n (%) | 3/6 (50) | 2/4 (50) |
| Neurological, n (%) | 41/64 (64) | 32/61 (52) |
| Psychiatric, n (%) | 93/141 (66) | 81/130 (62) |
| Respiratory, n (%) | 23/39 (59) | 18/38 (47) |
| Any comorbidity, n (%) | 298/533 (56) | 241/455 (53) |
| No comorbidity, n (%) | 290/555 (52) | 247/473 (52) |
| Age: 18-49 years | 168/325 (52) | 148/280 (53) |
| Age: 50-74 years | 395/714 (55) | 320/605 (53) |
| Age: ≥75 years | 25/49 (51) | 20/43 (47) |
| Seronegative | 195/362 (54) | 156/298 (52) |
| Seropositive | 387/712 (54) | 327/617 (53) |
| Males | 167/332 (50) | 129/281 (46) |
| Females | 421/756 (56) | 359/647 (55) |
| Educational level ≤9 years | 114/222 (51) | 111/199 (56) |
| Educational level: 10-12 years | 139/273 (51) | 130/233 (56) |
| Educational level: >12 years | 335/593 (56) | 247/496 (50) |
| Never smoker | 204/370 (55) | 171/317 (54) |
| Former smoker | 194/367 (53) | 150/301 (50) |
| Current smoker | 176/318 (55) | 151/277 (55) |
| Physical activity: inactive | 400/738 (54) | 349/633 (55) |
| Physical activity: active | 187/347 (54) | 137/292 (47) |
| No glucocorticoid treatment at initiation of MTX | 266/441 (60) | 199/363 (55) |
| Glucocorticoid treatment at initiation of MTX | 313/626 (50) | 278/546 (51) |

Missing information at three months: DAS28-remission: 197 (15.3%), serological status: 8 (0.7%), smoking: 19 (1.7%), physical activity 2 (0.1%), glucocorticoid treatment: 12 (1.1%). Missing information at six months: DAS28-remission: 357 (27.8%), serological status: 8 (0.8%), smoking: 17 (1.8%), physical activity: 1 (0.1%), glucocorticoid treatment: 8 (0.9%). Underweight: BMI <18.5 kg/m^2^, normal weight: BMI 18.5-24.9 kg/m^2^, overweight: BMI 25-29.9 kg/m^2^and obese: BMI ≥30 kg/m^2^. Physical activity, inactive: sedentary or moderate exercise, active: moderate regular exercise or regular exercise. DAS28, Disease Activity Score (28 joints); RDCI, Rheumatic Disease Comorbidity Index; MTX, methotrexate.

**Supplemental table S7.** Relative risk of not reaching DAS28-remission at three and six months among early RA patients receiving methotrexate monotherapy during 2006-2018, estimated by modified Poisson regression

|  | n with DAS28-remission at 3 months n=1088 | Not reaching DAS28-remission  at 3 months  (crude)RR (95% CI) | Not reaching DAS28-remission  at 3 months  (adjusted )  RR (95% CI) | Not reaching DAS28-remission at 3 months  (full)  RR (95% CI) | n with DAS28-remission at 6 months n=928 | Not reaching DAS28-remission  at 6 months  (crude)  RR (95% CI) | Not reaching DAS28-remission  at 6 months (adjusted)  RR (95% CI) | Not reaching DAS28-remission  at 6 months (full)  RR (95% CI) |
| --- | --- | --- | --- | --- | --- | --- | --- | --- |
| Normal weight | 505 | Reference | Reference | Reference | 433 | Reference | Reference | Reference |
| Overweight | 384 | 1.04 (0.92-1.19) | 1.05 (0.93-1.20) | 1.03 (0.91-1.17) | 326 | 1.12 (0.97-1.29) | 1.10 (0.96-1.27) | 1.09 (0.95-1.26) |
| Obese | 185 | **1.20 (1.04-1.38)** | **1.20 (1.04-1.38)** | 1.16 (1.00-1.34) | 153 | **1.33 (1.14-1.55)** | **1.29 (1.10-1.51)** | **1.27 (1.08-1.50)** |

Normal weight: BMI 18.5-24.9 kg/m^2^ (reference), overweight: BMI 25-29.9 kg/m^2^, obese: BMI ≥30 kg/m^2^. Crude: Adjusted for sex and age, Adjusted: Adjusted for serological status, glucocorticoid treatment at methotrexate initiation, educational level, smoking, alcohol use, physical activity and calendar period of methotrexate start, Full: Adjusted + the individual comorbidity categories. Statistically significant findings in bold. DAS28, Disease Activity Score (28 joints).

**Supplemental table S8.** Relative risk of not reaching DAS28-remission at three and six months, by BMI, sex, serological status and smoking, among early RA patients receiving methotrexate monotherapy during 2006-2018, estimated by modified Poisson regression

|  | n with data on DAS28-remission at 3 months | Not reaching DAS28-remission  at 3 months  (crude)  RR (95% CI) | Not reaching DAS28-remission  at 3 months  (adjusted )  RR (95% CI) | Not reaching DAS28-remission at 3 months  (full)  RR (95% CI) | n with data on DAS28-remission at 6 months | Not reaching DAS28-remission  at 6 months  (crude)  RR (95% CI) | Not reaching DAS28-remission  at 6 months (adjusted)  RR (95% CI) | Not reaching DAS28-remission  at 6 months (full)  RR (95% CI) |
| --- | --- | --- | --- | --- | --- | --- | --- | --- |
| Men, n | 332 | 332 | 332 | 332 | 281 | 281 | 281 | 281 |
| Normal weight | 126 | Reference | Reference | Reference | 108 | Reference | Reference | Reference |
| Overweight | 151 | 1.00 (0.79-1.27) | 1.00 (0.78-1.27) | 0.96 (0.75-1.22) | 130 | 1.11 (0.83-1.48) | 1.06 (0.79-1.41) | 1.01 (0.75-1.36) |
| Obese | 55 | 0.96 (0.70-1.31) | 0.94 (0.68-1.30) | 0.85 (0.61-1.20) | 43 | 1.25 (0.89-1.77) | 1.15 (0.81-1.63) | 1.08 (0.75-1.55) |
| Women, n | 756 | 756 | 756 | 756 | 647 | 647 | 647 | 647 |
| Normal weight | 379 | Reference | Reference | Reference | 325 | Reference | Reference | Reference |
| Overweight | 233 | 1.05 (0.90-1.22) | 1.08 (0.93-1.25) | 1.08 (0.93-1.26) | 196 | 1.11 (0.95-1.31) | 1.14 (0.97-1.35) | 1.16 (0.98-1.37) |
| Obese | 130 | **1.28 (1.09-1.49)** | **1.30 (1.11-1.52)** | **1.26 (1.08-1.49)** | 110 | **1.35 (1.14-1.59)** | **1.35 (1.14-1.61)** | **1.36 (1.13-1.62)** |
| Seronegative, n | 358 | 358 | 358 | 358 | 298 | 298 | 298 | 298 |
| Normal weight | 191 | Reference | Reference | Reference | 141 | Reference | Reference | Reference |
| Overweight | 132 | 1.06 (0.85-1.32) | 1.04 (0.84-1.29) | 1.03 (0.83-1.28) | 104 | 1.20 (0.94-1.54) | 1.17 (0.9-1.5) | 1.14 (0.89-1.47) |
| Obese | 55 | 1.17 (0.90-1.51) | 1.09 (0.84-1.41) | 0.98 (0.76-1.28) | 48 | 1.33 (1.01-1.74) | 1.23 (0.93-1.62) | 1.23 (0.93-1.64) |
| Seropositive, n | 712 | 712 | 712 | 712 | 617 | 617 | 617 | 617 |
| Normal weight | 324 | Reference | Reference | Reference | 284 | Reference | Reference | Reference |
| Overweight | 248 | 1.05 (0.89-1.22) | 1.06 (0.91-1.24) | 1.03 (0.88-1.2) | 219 | 1.10 (0.92-1.31) | 1.07 (0.90-1.27) | 1.06 (0.89-1.26) |
| Obese | 130 | **1.21 (1.02-1.43)** | **1.23 (1.03-1.46)** | 1.19 (1.00-1.42) | 103 | **1.32 (1.09-1.59)** | **1.26 (1.04-1.53)** | **1.25 (1.02-1.53)** |
| Non-smokers*, n | 318 | 318 | 318 | 318 | 277 | 277 | 277 | 277 |
| Normal weight | 165 | Reference | Reference | Reference | 145 | Reference | Reference | Reference |
| Overweight | 104 | 1.1 (0.88-1.37) | 1.07 (0.87-1.33) | 1.05 (0.85-1.31) | 89 | 0.95 (0.73-1.23) | 0.93 (0.73-1.20) | 0.93 (0.72-1.20) |
| Obese | 43 | **1.37 (1.07-1.76)** | 1.27 (0.99-1.64) | 1.15 (0.88-1.51) | 37 | 1.27 (0.97-1.67) | 1.20 (0.90-1.60) | 1.10 (0.80-1.50) |

| Smokers**, n | 737 | 737 | 737 | 737 | 618 | 618 | 618 | 618 |
| --- | --- | --- | --- | --- | --- | --- | --- | --- |
| Normal weight | 324 | Reference | Reference | Reference | 273 | Reference | Reference | Reference |
| Overweight | 266 | 1.04 (0.89-1.22) | 1.05 (0.89-1.23) | 1.03 (0.88-1.21) | 223 | **1.25 (1.05-1.50)** | **1.23 (1.03-1.48)** | **1.24 (1.03-1.49)** |
| Obese | 140 | 1.14 (0.96-1.36) | 1.17 (0.99-1.40) | 1.15 (0.96-1.37) | 113 | **1.40 (1.16-1.69)** | **1.37 (1.12-1.66)** | **1.36 (1.11-1.67)** |
| Obese vs non-obese, n | 1088 | 1088 | 1088 | 1088 | 928 | 928 | 928 | 928 |
| Non-obese | 903 | Reference | Reference | Reference | 775 | Reference | Reference | Reference |
| Obese vs non-obese | 185 | **1.17 (1.03-1.34)** | **1.17 (1.03-1.33)** | 1.14 (1.00-1.30) | 153 | **1.27 (1.10-1.45)** | **1.23 (1.07-1.41)** | **1.22 (1.05-1.41)** |

*Non-smokers: never-smokers. **Smokers: current and former smokers. Normal weight: BMI 18.5-24.9 kg/m^2^, overweight: BMI 25-29.9 kg/m^2^, obese: BMI ≥30 kg/m^2^. Normal weight is the reference. Crude: Adjusted for sex and age, Adjusted: Adjusted for serological status, glucocorticoid treatment at methotrexate initiation (yes/no), educational level, smoking (never[ref]/past/current, alcohol use, physical activity (active [ref]/inactive) and calendar period of methotrexate start (2006-2014, 2015-2018), Full: Adjusted + the individual comorbidity categories. Statistically significant findings in bold. DAS28, Disease Activity Score (28 joints); BMI, body mass index.

**Supplemental table S9.** Relative risk of not reaching DAS28-remission by comorbidity category, for each BMI category separately, among early RA patients receiving methotrexate monotherapy during 2006-2018, estimated by modified Poisson regression

|  | DAS28-remission  at 3 months (crude)  RR (95% CI) | DAS28-remission at 3 months (adjusted)  RR (95% CI) | DAS28-remission at 3 months  (full)  RR (95% CI) | DAS28-remission at 6 months (crude)  RR (95% CI) | DAS28-remission at 6 months (adjusted)  RR (95% CI) | DAS28-remission at 6 months  (full)  RR (95% CI) |
| --- | --- | --- | --- | --- | --- | --- |
| Normal weight, n | 505 | 505 | 505 | 433 | 433 | 433 |
| Cardiovascular | **1.50 (1.15-1.96)**** | **1.50 (1.15-1.94)**** | **1.39 (1.03-1.89)*** | 1.33 (0.86-2.06) | 1.34 (0.85-2.12) | 1.61 (0.94-2.76) |
| Non-cardiac vascular | 1.22 (0.98-1.53) | **1.28 (1.02-1.59)*** | 1.18 (0.92-1.50) | 0.97 (0.72-1.32) | 0.98 (0.72-1.34) | 0.84 (0.59-1.20) |
| Malignant | 0.72 (0.41-1.24) | 0.72 (0.43-1.21) | 0.66 (0.40-1.09) | 0.67 (0.30-1.51) | 0.70 (0.31-1.61) | 0.66 (0.30-1.45) |
| Endocrine | 0.84 (0.62-1.13) | 0.81 (0.60-1.09) | 0.77 (0.58-1.03) | 0.80 (0.55-1.17) | 0.84 (0.57-1.25) | 0.84 (0.56-1.24) |
| Gastrointestinal | 1.35 (0.93-1.96) | 1.22 (0.84-1.78) | 1.25 (0.86-1.83) | 0.80 (0.40-1.61) | 0.74 (0.36-1.50) | 0.68 (0.32-1.45) |
| Infectious | 1.31 (0.96-1.78) | 1.26 (0.94-1.67) | 1.21 (0.89-1.64) | 0.79 (0.46-1.38) | 0.74 (0.44-1.26) | 0.77 (0.45-1.32) |
| Chronic kidney diseaseƗ | - | - | - | - | - | - |
| Neurological | 1.18 (0.88-1.59) | 1.16 (0.87-1.55) | 1.12 (0.84-1.5) | 0.97 (0.65-1.46) | 0.97 (0.65-1.43) | 0.94 (0.63-1.42) |
| Psychiatric | 1.14 (0.89-1.45) | 1.17 (0.92-1.49) | 1.14 (0.90-1.45) | **1.36 (1.08-1.72)**** | **1.38 (1.10-1.75)**** | **1.42 (1.12-1.80)**** |
| Respiratory | 0.99 (0.45-2.18) | 0.99 (0.49-2.03) | 0.91 (0.47-1.78) | 1.01 (0.50-2.05) | 1.11 (0.58-2.12) | 1.16 (0.64-2.10) |
| Overweight, n | 384 | 384 | 384 | 326 | 326 | 326 |
| Cardiovascular | 1.17 (0.82-1.66) | 1.21 (0.86-1.72) | 1.29 (0.89-1.88) | 1.33 (0.91-1.93) | 1.33 (0.91-1.95) | **1.54 (1.06-2.23)*** |
| Non-cardiac vascular | 0.98 (0.78-1.23) | 0.97 (0.77-1.23) | 0.91 (0.72-1.16) | 1.00 (0.77-1.3) | 0.96 (0.74-1.25) | 0.91 (0.69-1.20) |
| Malignant | **1.53 (1.12-2.08)**** | **1.54 (1.10-2.17)*** | **1.61 (1.14-2.26)**** | 1.18 (0.72-1.96) | 1.23 (0.73-2.09) | 1.40 (0.88-2.24) |
| Endocrine | 1.05 (0.82-1.33) | 1.04 (0.82-1.32) | 1.00 (0.79-1.28) | 1.09 (0.85-1.40) | 1.05 (0.81-1.35) | 1.08 (0.84-1.40) |
| Gastrointestinal | 0.93 (0.59-1.45) | 0.90 (0.57-1.43) | 0.96 (0.61-1.53) | 0.91 (0.55-1.51) | 0.97 (0.58-1.63) | 0.99 (0.60-1.64) |
| Infectious | 1.16 (0.79-1.71) | 1.15 (0.77-1.72) | 1.10 (0.75-1.63) | 0.52 (0.22-1.24) | 0.49 (0.21-1.12) | 0.45 (0.18-1.12) |
| Chronic kidney diseaseƗ | - | - | - | - | - | - |
| Neurological | **1.45 (1.11-1.89)**** | **1.49 (1.13-1.96)**** | **1.51 (1.13-2.01)**** | 0.91 (0.59-1.41) | 0.95 (0.62-1.48) | 1.00 (0.63-1.59) |
| Psychiatric | **1.36 (1.11-1.69)**** | **1.36 (1.09-1.69)**** | **1.36 (1.10-1.69)**** | 1.10 (0.85-1.44) | 1.08 (0.83-1.42) | 1.07 (0.82-1.39) |
| Respiratory | 1.09 (0.76-1.57) | 1.08 (0.75-1.56) | 1.05 (0.72-1.53) | 0.86 (0.52-1.43) | 0.80 (0.48-1.31) | 0.74 (0.45-1.22) |

| Obese, n | 185 | 185 | 185 | 153 | 153 | 153 |
| --- | --- | --- | --- | --- | --- | --- |
| Cardiovascular | 0.99 (0.65-1.51) | 1.03 (0.68-1.56) | 0.93 (0.6-1.45) | 0.62 (0.28-1.38) | 0.61 (0.28-1.33) | 0.66 (0.31-1.40) |
| Non-cardiac vascular | 1.08 (0.84-1.41) | 1.14 (0.88-1.47) | 1.13 (0.86-1.49) | 1.05 (0.79-1.39) | 0.99 (0.75-1.32) | 1.03 (0.78-1.36) |
| Malignant | 0.52 (0.21-1.30) | 0.55 (0.22-1.35) | 0.50 (0.20-1.26) | 0.93 (0.56-1.54) | 0.91 (0.54-1.54) | 0.83 (0.48-1.42) |
| Endocrine | 1.18 (0.92-1.50) | 1.15 (0.90-1.47) | 1.15 (0.90-1.46) | **1.34 (1.06-1.69)*** | **1.35 (1.06-1.73)*** | **1.39 (1.08-1.79)*** |
| Gastrointestinal | 1.23 (0.91-1.68) | 1.32 (0.91-1.92) | 1.36 (0.94-1.95) | 0.98 (0.57-1.68) | 0.93 (0.54-1.61) | 0.92 (0.51-1.65) |
| Infectious | 0.93 (0.57-1.49) | 0.90 (0.55-1.47) | 0.82 (0.49-1.36) | 0.90 (0.52-1.55) | 0.93 (0.52-1.65) | 1.07 (0.59-1.93) |
| Chronic kidney diseaseƗ | - | - | - | - | - | - |
| Neurological | 0.85 (0.51-1.42) | 0.88 (0.53-1.45) | 0.94 (0.56-1.58) | 1.02 (0.66-1.57) | 1.03 (0.64-1.64) | 1.23 (0.80-1.87) |
| Psychiatric | 1.17 (0.91-1.49) | 1.17 (0.91-1.5) | 1.23 (0.96-1.57) | 0.96 (0.72-1.27) | 0.91 (0.68-1.22) | 1.00 (0.74-1.35) |
| Respiratory | 1.16 (0.74-1.81) | 1.17 (0.75-1.85) | 1.02 (0.64-1.63) | 0.89 (0.48-1.65) | 0.87 (0.46-1.66) | 0.81 (0.41-1.60) |

*P-values <0.05. **P-values <0.01. ƗThere were ≤5 patients with Chronic kidney disease in each BMI category and results were not calculated for this comorbid condition. Normal weight: BMI 18.5-24.9 kg/m^2^, overweight: BMI 25-29.9 kg/m^2^ and obese: BMI ≥30 kg/m^2^. Normal weight is the reference. Crude: Adjusted for sex and age, Adjusted: Adjusted for serological status, glucocorticoid treatment at methotrexate initiation (yes/no), educational level, smoking (never[ref]/past/current, alcohol use, physical activity (active [ref]/inactive) and calendar period of methotrexate start (2006-2014, 2015-2018), Full: Adjusted + the individual comorbidity categories. Statistically significant findings in bold. DAS28, Disease Activity Score (28 joints); BMI, body mass index.

**Supplemental table S10.** Relative risk of not reaching Boolean, CDAI, SDAI remission, EULAR response and no swollen joints in patients with overweight and obesity at three and six months, estimated by modified Poisson regression

| Boolean remission | n with data on Boolean remission at 3 months n=1083 | Not  reaching Boolean remission at 3 months  (crude)  RR (95% CI) | Not  reaching Boolean remission at 3  months (adjusted)  RR (95% CI) | Not  reaching Boolean remission at 3 months  (full)  RR (95% CI) | n with data on Boolean remission at 6 months n=922 | Not  reaching Boolean remission at 6  months  (crude)  RR (95% CI) | Not  reaching Boolean remission at 6 months (adjusted)  RR (95% CI) | Not  reaching Boolean remission at 6 months  (full)  RR (95% CI) |
| --- | --- | --- | --- | --- | --- | --- | --- | --- |
| Normal weight | 503 | Reference | Reference | Reference | 431 | Reference | Reference | Reference |
| Overweight | 383 | 1.03 (0.96-1.10) | 1.03 (0.96-1.10) | 1.01 (0.95-1.09) | 323 | 1.06 (0.98-1.15) | 1.05 (0.97-1.14) | 1.03 (0.95-1.12) |
| Obese | 183 | **1.10 (1.02-1.18)** | **1.09 (1.01-1.17)** | 1.06 (0.98-1.14) | 152 | **1.17 (1.08-1.28)** | **1.15 (1.06-1.26)** | **1.12 (1.02-1.23)** |
| CDAI remission | n with data on CDAI remission at 3 months n=1055 | Not  reaching  CDAI remission at 3 months (crude)  RR (95% CI) | Not  reaching  CDAI remission at 3 months (adjusted)  RR (95% CI) | Not  reaching  CDAI remission at 3 months (full)  RR (95% CI) | n with data on CDAI remission at 6 months  n=919 | Not  reaching  CDAI remission at 6 months (crude)  RR (95% CI) | Not  reaching  CDAI remission at 6 months (adjusted)  RR (95% CI) | Not  reaching  CDAI remission at 6 months (full)  RR (95% CI) |
| Normal weight | 490 | Reference | Reference | Reference | 428 | Reference | Reference | Reference |
| Overweight | 369 | 1.04 (0.97-1.12) | 1.05 (0.97-1.13) | 1.04 (0.96-1.11) | 325 | 1.04 (0.96-1.13) | 1.04 (0.96-1.13) | 1.03 (0.94-1.11) |
| Obese | 183 | **1.10 (1.01-1.19)** | 1.09 (1.00-1.18) | 1.06 (0.98-1.15) | 150 | **1.13 (1.04-1.24)** | **1.14 (1.04-1.25)** | **1.12 (1.02-1.23)** |
| SDAI remission | n with data on SDAI remission at 3 months  n=1054 | Not reaching SDAI remission at 3 months (crude)  RR (95% CI) | Not reaching SDAI remission at 3 months (adjusted)  RR (95% CI) | Not reaching SDAI remission at 3 months (full)  RR (95% CI) | n with data on SDAI remission at 6 months n=906 | Not reaching SDAI remission at 6 months  (crude)  RR (95% CI) | Not reaching SDAI remission at 6 months (adjusted)  RR (95% CI) | Not reaching SDAI remission at 6 months (full)  RR (95% CI) |
| Normal weight | 490 | Reference | Reference | Reference | 424 | Reference | Reference | Reference |
| Overweight | 370 | 1.03 (0.95-1.11) | 1.04 (0.96-1.12) | 1.03 (0.95-1.11) | 319 | 1.05 (0.96-1.15) | 1.05 (0.96-1.15) | 1.04 (0.95-1.14) |
| Obese | 180 | **1.09 (1.01-1.19)** | **1.10 (1.01-1.20)** | 1.07 (0.98-1.17) | 147 | **1.17 (1.06-1.28)** | **1.17 (1.06-1.29)** | **1.15 (1.04-1.28)** |
| EULAR response | n with data on EULAR response at 3 months n=1028 | Not reaching EULAR response at 3 months (crude)  RR (95% CI) | Not reaching EULAR response at 3 months (adjusted)  RR (95% CI) | Not reaching EULAR response at 3 months (full)  RR (95% CI) | n with data on EULAR response at 6 months n=877 | Not reaching EULAR response at 6 months (crude)  RR (95% CI) | Not reaching EULAR response at 6 months (adjusted)  RR (95% CI) | Not reaching EULAR response at 6 months (full)  RR (95% CI) |
| Normal weight | 476 | Reference | Reference | Reference | 409 | Reference | Reference | Reference |
| Overweight | 360 | 1.08 (0.93-1.27) | 1.10 (0.94-1.29) | 1.11 (0.95-1.29) | 307 (35) | 1.12 (0.94-1.33) | 1.09 (0.91-1.29) | 1.09 (0.92-1.30) |
| Obese | 178 | **1.25 (1.06-1.48)** | **1.24 (1.04-1.47)** | **1.25 (1.04-1.49)** | 145 (16) | **1.27 (1.05-1.54)** | 1.19 (0.97-1.45) | 1.21 (0.99-1.48) |
| No swollen joints | n with data on swollen joints  at 3 months n=1107 | No swollen joints at 3 months  (crude)  RR (95% CI) | No swollen joints at 3 months (adjusted )  RR (95% CI) | No swollen joints at 3 months  (full)  RR (95% CI) | n with data on swollen joints  at 6 months n=945 | No swollen joints at 6  months  (crude)  RR (95% CI) | No swollen joints at 6 months (adjusted)  RR (95% CI) | No swollen joints at 6 months  (full)  RR (95% CI) |
| Normal weight | 512 | Reference | Reference | Reference | 439 | Reference | Reference | Reference |
| Overweight | 391 | 1.04 (0.89-1.21) | 1.03 (0.88-1.20) | 1.06 (0.91-1.24) | 335 | 0.99 (0.85-1.16) | 1.01 (0.87-1.18) | 1.03 (0.88-1.21) |
| Obese | 190 | 1.05 (0.86-1.27) | 1.04 (0.86-1.27) | 1.10 (0.90-1.35) | 155 | 0.90 (0.73-1.11) | 0.91 (0.73-1.12) | 0.93 (0.75-1.16) |

Normal weight: BMI 18.5-24.9 kg/m^2^, overweight: BMI 25-29.9 kg/m^2^, obese: BMI ≥30 kg/m^2^. Normal weight is the reference. Crude: Adjusted for sex and age, Adjusted: Adjusted for serological status, glucocorticoid treatment at methotrexate initiation (yes/no), educational level, smoking (never[ref]/past/current), alcohol use, physical activity (active [ref]/inactive) and calendar period of methotrexate start (2006-2014, 2015-2018), Full: Adjusted + the individual comorbidity categories. Statistically significant findings in bold. CDAI, Clinical Disease Activity Index; SDAI, Simplified Disease Activity Index; EULAR response, European Alliance of Associations for Rheumatology good response compared to moderate/no response. No swollen joint count was calculated on 28 joints.

**Supplemental table S11.** Relative risk of CRP >10 mg/l, ESR >15/20* mm/h, VAS Pain >20 mm, Patient Global Assessment >20 mm, Swollen Joint Count >1 and Tender Joint Count >1 in patients with overweight and obesity at three and six months, estimated by modified Poisson regression

| CRP >10 mg/l | n with data on CRP at 3 months  n=1102 | CRP >10 mg/l  at 3 months (crude)  RR (95% CI) | CRP >10 mg/l  at 3 months (adjusted)  RR (95% CI) | CRP >10 mg/l  at 3 months  (full)  RR (95% CI) | n with data on CRP at 6 months n=974 | CRP >10 mg/l  at 6 months (crude)  RR (95% CI) | CRP >10 mg/l  at 6 months (adjusted)  RR (95% CI) | CRP >10 mg/l  at 6 months (full)  RR (95% CI) |
| --- | --- | --- | --- | --- | --- | --- | --- | --- |
| Normal weight | 511 | Reference | Reference | Reference | 460 | Reference | Reference | Reference |
| Overweight | 390 | 1.34 (0.99-1.81) | **1.37 (1.01-1.86)** | **1.41 (1.04-1.93)** | 342 | 1.36 (0.95-1.96) | 1.28 (0.89-1.84) | 1.32 (0.92-1.89) |
| Obese | 187 | **1.50 (1.05-2.14)** | **1.61 (1.12-2.31)** | **1.66 (1.15-2.39)** | 156 | 1.53 (0.99-2.35) | 1.42 (0.91-2.21) | 1.49 (0.95-2.33) |
| ESR >15/20 mm/h* | n with data on ESR at 3 months n=1035 | ESR >15/20  mm/h*  at 3 months (crude)  RR (95% CI) | ESR >15/20 mm/h*  at 3 months (adjusted)  RR (95% CI) | ESR >15/20  mm/h*  at 3 months  (full)  RR (95% CI) | n with data on ESR at 6 months  n=946 | ESR >15/20  mm/h*  at 6 months (crude)  RR (95% CI) | ESR >15/20  mm/h*  at 6 months  (adjusted)  RR (95% CI) | ESR >15/20  mm/h*  at 6 months (full)  RR (95% CI) |
| Normal weight | 476 | Reference | Reference | Reference | 448 | Reference | Reference | Reference |
| Overweight | 363 | **1.26 (1.05-1.52)** | **1.24 (1.03-1.49)** | **1.24 (1.02-1.50)** | 332 | 1.18 (0.93-1.50) | 1.14 (0.90-1.45) | 1.13 (0.88-1.44) |
| Obese | 185 | **1.39 (1.11-1.73)** | **1.33 (1.06-1.67)** | **1.33 (1.05-1.68)** | 152 | **1.88 (1.47-2.41)** | **1.77 (1.37-2.29)** | **1.73 (1.33-2.25)** |
| VAS Pain >20 mm | n with data on VAS Pain at 3 months n=1092 | VAS Pain >20 mm  at 3 months (crude)  RR (95% CI) | VAS Pain >20 mm  at 3 months (adjusted)  RR (95% CI) | VAS Pain >20 mm  at 3 months  (full)  RR (95% CI) | n with data on VAS Pain at 6 months n=973 | VAS Pain >20 mm  at 6 months (crude)  RR (95% CI) | VAS Pain >20 mm  at 6 months (adjusted)  RR (95% CI) | VAS Pain >20 mm  at 6 months (full)  RR (95% CI) |
| Normal weight | 508 | Reference | Reference | Reference | 461 | Reference | Reference | Reference |
| Overweight | 384 | **1.32 (1.15-1.52)** | **1.32 (1.15-1.51)** | **1.28 (1.11-1.47)** | 342 | 1.15 (1.00-1.32) | 1.13 (0.98-1.30) | 1.09 (0.95-1.26) |
| Obese | 186 | **1.33 (1.13-1.57)** | **1.27 (1.07-1.50)** | 1.19 (1.00-1.41) | 154 | 1.14 (0.96-1.36) | 1.11 (0.93-1.34) | 1.07 (0.89-1.29) |
| PGA >20 mm | n with data on PGA  at 3 months n=1095 | PGA >20 mm  at 3 months (crude)  RR (95% CI) | PGA >20 mm  at 3 months (adjusted)  RR (95% CI) | PGA >20 mm  at 3 months (full)  RR (95% CI) | n with data on PGA  at 6 months n=979 | PGA >20 mm  at 6 months (crude)  RR (95% CI) | PGA >20 mm  at 6 months (adjusted)  RR (95% CI) | PGA >20 mm  at 6 months (full)  RR (95% CI) |
| Normal weight | 510 | Reference | Reference | Reference | 463 | Reference | Reference | Reference |
| Overweight | 384 | **1.23 (1.08-1.40)** | **1.22 (1.07-1.39)** | **1.19 (1.04-1.35)** | 342 | 1.14 (0.99-1.31) | 1.13 (0.98-1.29) | 1.09 (0.94-1.25) |
| Obese | 188 | **1.34 (1.16-1.55)** | **1.27 (1.10-1.47)** | **1.20 (1.03-1.40)** | 158 | **1.33 (1.14-1.54)** | **1.30 (1.11-1.52)** | **1.24 (1.05-1.46)** |
| SJC >1 | n with data on SJC at 3 months n=1111 | SJC >1  at 3 months (crude)  RR (95% CI) | SJC >1  at 3 months (adjusted)  RR (95% CI) | SJC >1  at 3 months  (full)  RR (95% CI) | n with data on SJC at 6 months n=987 | SJC >1  at 6 months (crude)  RR (95% CI) | SJC >1  at 6 months (adjusted)  RR (95% CI) | SJC >1  at 6 months (full)  RR (95% CI) |
| Normal weight | 516 | Reference | Reference | Reference | 462 | Reference | Reference | Reference |
| Overweight | 391 | 0.95 (0.81-1.10) | 0.95 (0.82-1.11) | 0.93 (0.79-1.08) | 349 | 1.05 (0.86-1.28) | 1.04 (0.85-1.26) | 1.02 (0.83-1.24) |
| Obese | 190 | 0.91 (0.74-1.10) | 0.91 (0.74-1.11) | 0.86 (0.70-1.06) | 160 | 1.02 (0.79-1.31) | 1.01 (0.78-1.30) | 0.97 (0.75-1.27) |
| TJC >1 | n with data on TJC at 3 months n=1111 | TJC >1  at 3 months (crude)  RR (95% CI) | TJC >1  at 3 months (adjusted)  RR (95% CI) | TJC >1  at 3 months (full)  RR (95% CI) | n with data on TJC at 6 months n=987 | TJC >1  at 6 months (crude)  RR (95% CI) | TJC >1  at 6 months (adjusted)  RR (95% CI) | TJC >1  at 6 months (full)  RR (95% CI) |
| Normal weight | 516 | Reference | Reference | Reference | 463 | Reference | Reference | Reference |
| Overweight | 391 | 0.98 (0.84-1.14) | 1.00 (0.86-1.16) | 0.97 (0.84-1.13) | 349 | 1.15 (0.96-1.37) | 1.13 (0.94-1.35) | 1.11 (0.92-1.32) |
| Obese | 190 | 1.15 (0.97-1.35) | 1.18 (1.00-1.40) | 1.11 (0.93-1.32) | 159 | 1.20 (0.97-1.48) | 1.15 (0.92-1.43) | 1.12 (0.89-1.40) |

*ESR >20 mm/h in women and >15 mm/h in men. Normal weight: BMI 18.5-24.9 kg/m^2^, overweight: BMI 25-29.9 kg/m^2^, obese: BMI ≥30 kg/m^2^. Normal weight is the reference. Crude: Adjusted for sex and age. Adjusted: Adjusted for serological status, glucocorticoid treatment at methotrexate initiation (yes/no), educational level, smoking (never[ref]/past/current), alcohol use, physical activity (active [ref]/inactive) and calendar period of methotrexate start (2006-2014, 2015-2018). Full: Adjusted + the individual comorbidity categories. Statistically significant findings in bold. CRP, C-reactive protein; ESR, erythrocyte sedimentation rate; VAS, Visual Analogue Scale in mm; PGA, Patient Global Assessment; SJC, Swollen Joint Count (28 joints); TJC, Tender Joint Count (28 joints).
